# Boredom at the border of philosophy: conceptual and ethical issues

**DOI:** 10.3389/fsoc.2023.1178053

**Published:** 2023-07-10

**Authors:** Peter Levine

**Affiliations:** Tisch College of Civic Life and Departments of Philosophy and Political Science, Tufts University, Medford, MA, United States

**Keywords:** boredom, phenomenology, Heidegger, Adorno, normative issues in measurement

## Abstract

Boredom is a topic in philosophy. Philosophers have offered close descriptions of the experience of boredom that should inform measurement and analysis of empirical results. Notable historical authors include Seneca, Martin Heidegger, and Theodor Adorno; current philosophers have also contributed to the literature. Philosophical accounts differ in significant ways, because each theory of boredom is embedded in a broader understanding of institutions, ethics, and social justice. Empirical research and interventions to combat boredom should be conscious of those frameworks. Philosophy can also inform responses to normative questions, such as whether and when boredom is bad and whether the solution to boredom should involve changing the institutions that are perceived as boring, the ways that these institutions present themselves, or individuals' attitudes and choices.

## 1. Introduction: why philosophy is relevant

Whether boredom leads to consequences such as violence is an empirical question, as is the question of whether boredom can be prevented in various ways. Other contributions to this volume consider those issues. I presume that the harmful consequences of boredom are serious and deserve both empirical research and effective interventions.

Boredom is also a topic in philosophy (Svendsen, 2005; Toohey, 2011; O'Brien, 2014, 2022; Elpidorou, 2020). Seneca, Martin Heidegger, and Theodor Adorno are among the historical philosophers who addressed boredom in some detail. There is also now a substantial twenty-first century literature.

This article is intended as a brief review of the philosophical literature. I refer to some empirical psychology that is directly relevant to philosophical claims, without attempting to summarize the substantial psychological literature. I also argue that two philosophical issues are relevant to research on boredom and to interventions to prevent boredom or to address its consequences.

First, the proper definition of boredom is not simply an empirical question. There can be debate about whether one's own state or someone else's reflects weariness, frustration, impatience, apathy, depression, languor, and even tranquility or calm. Interview subjects would not all define it the same way. Indeed, it is possible to be bored without realizing it (Svendsen, 2005, p. 14). Since boredom comes in many forms, its defining features are not immediately evident. Phenomenology (a philosophical method) is suited to defining boredom, which is necessary for measuring boredom and investigating its causes and consequences. Specifically, phenomenology can help to distinguish between boredom as a problematic subjective state vs. simply not doing something notable—for instance, while being laid off during a pandemic.

An overview of survey measures finds “some common themes among the definitions of boredom,” which “include an understimulating environment, attention issues, perception of time passing slowly, insufficient challenge and meaning, and the pairing of low arousal with dissatisfaction” (Vodanovich and Watt, 2016). These themes overlap with the philosophical literature on boredom—but incompletely. Some of the concrete survey items in the Multidimensional State Boredom Scale (Fahlman et al., 2013) would make sense to philosophers whose work is discussed later. For example, “Time is passing by slower than usual” captures a central feature of Heidegger's account of boredom (Heidegger, 1995, §23a). But other items, such as “I feel agitated” or “I am lonely,” are not aspects of definitions of boredom that appear in the philosophical literature. These items contribute to a psychometrically valid scale, but that does not prove that they help indicate a trait or state that should be labeled as “boredom.” To assess that claim requires conceptual work.

Second, we need an informed view of normative or ethical questions, such as whether and when boredom is intrinsically undesirable (a bad way to be, not just a cause of bad outcomes), whether bored people should change their circumstances and activities or else change their attitudes toward what they are doing, and whether individuals or contexts and institutions are responsible for people being bored.

For the purposes of this volume and project, it is appropriate to define boredom as an undesirable, undesired, and unpleasant state. This premise is also consistent with some philosophical analyses of the experience of boredom (O'Brien, 2014). For some contemporary experts, boredom is a “negative feeling” that “operates as a positive signal” (Danckert and Eastwood, 2020; cf. Elpidorou, 2020). On that view, boredom is intrinsically undesirable but has valuable consequences. Some sources go further and at least hint at the intrinsic value of boredom (Toohey, 2011). The critic Walter Benjamin wrote:

If sleep is the pinnacle of physical relaxation, so boredom [*Langeweile*] is for the mind. Boredom is the dreambird that incubates the egg of experience. The rustling in the forest of leaves drives him away. Its roosts—the activities that are intimately associated with boredom—have already died out in the cities and are also dying out in the country. With that, the gift of listening is lost, and the community of listeners disappears (Benjamin, 1977).

Likewise, Heidegger's famous (and 89-page-long) analysis presents profound boredom as a door to fundamental truths and an opportunity to discover one's existential freedom (Heidegger, 1995, §19–38; Slaby, 2010; Freeman and Andreas, 2015; Elpidorou and Freeman, 2019).

The reason that we (contributors to this special issue) define boredom as undesirable is normative. The research collected here is a purposive activity for public good, addressing boredom as a problem. Such purposes are not biases that distort research; they are integral to valuable research. Normative assumptions can be defended, but they need explicit critical analysis.

Benjamin, as a heterodox Marxist literary critic, had values and goals that encouraged his positive view of boredom. He was seeking alternatives to the busyness of commercial capitalism. What Heidegger wanted to accomplish in 1930 (3 years before he joined the Nazi Party) is controversial, but his discussion of boredom includes remarks like this: “We should not be at all surprised if the contemporary man in the street feels disturbed or perhaps sometimes dazed … We must first call for someone capable of instilling terror into our Dasein [roughly: human experience] again” (Heidegger, 1995, §39; Hunt, 1998). Heidegger is accountable for his normative principles, as is anyone who studies a topic of human importance. The question is whether our principles are good (Levine, 2022, p. 46–53).

Specifically, it is worth asking whether boredom is intrinsically undesirable or wrong, not merely linked to bad outcomes (or good ones, such as realizing that one's current activity is meaningless). One reason to ask this question is existential: we should investigate how to live well as individuals. Are we obliged not to be bored? Another reason is more pragmatic. If being bored is wrong, we might look for effective ways to express that fact, which might influence people's behaviors. For instance, children are often scolded for being bored. If being bored is not wrong, then we shouldn't—and probably cannot—change behavior by telling people that it's wrong to be bored. Relatedly, when is it a valid critique of an organization or institution to claim that it causes boredom or is boring? Might it be necessary and appropriate for some institutions (such as the Federal Reserve) to be boring?

Just as social and behavioral sciences need philosophy (especially phenomenology and normative analysis), so philosophy needs the social sciences. Statistical questions, such as the prevalence of boredom and its association with depression or violence, should influence our definitions and evaluations of boredom. Phenomenology can degenerate into social science with an unrepresentative sample of one, which has serious drawbacks for generalizability. Therefore, the influence of philosophy and social science should be reciprocal. We need general patterns plus close introspective analysis. This paper introduces prominent ideas from the philosophical literature.

## 2. Phenomenology of boredom

The classic method of phenomenology is the close, explicit description of an inner state that avoids (or “brackets”) as many theoretical preconceptions as possible. Husserl writes, “phenomenological explication does nothing but explicate the sense this world has for us all, prior to any philosophizing” (Husserl, 1929, § 62). A phenomenological account is meant to ring true to its reader. A related method is to analyze and interpret descriptions of other people's experiences as they have been presented in history, autobiography, and fiction. For instance, Goodstein's *Experience Without Qualities* (Goodstein, 2005) is mostly a set of close readings of literary descriptions of boredom. Goodstein is a critic rather than an introspective phenomenologist, but the two methods are similar. Empirical psychologists like James Danckert and John D. Eastwood also read, interpret and directly practice phenomenology (Danckert and Eastwood, 2020).

Perhaps the first phenomenology of boredom appeared before 62 CE in a letter by the Stoic philosopher Seneca. He describes a person who is dissatisfied with himself, driven to play ambitious roles in public life (where he suffers inevitable failures), regrets his defeats, and then

[retreats] to idleness and to secret studies, which are unendurable to a mind eager to take part in public affairs, desirous of action and naturally restless, because, of course, it finds too few resources within itself: when therefore it loses the amusement which business itself affords to busy men, it cannot endure home, loneliness, or the walls of a room, and regards itself with dislike when left to itself. Hence arises that weariness [*taedium*] and dissatisfaction with oneself, that tossing to and fro of a mind which can nowhere find rest, that unhappy and unwilling endurance of enforced leisure. … Hence comes melancholy and drooping of spirit, and a thousand waverings of the unsteadfast mind, which is held in suspense by unfulfilled hopes, and saddened by disappointed ones: hence comes the state of mind of those who loathe their idleness [*otium*: vacant time], complain that they have nothing to do, and view the progress of others with the bitterest jealousy: for an unhappy sloth favors the growth of envy, and men who cannot succeed themselves wish everyone else to be ruined. This dislike of other men's progress and despair of one's own produces a mind angered against fortune, addicted to complaining of the age in which it lives to retiring into corners and brooding over its misery, until it becomes sick and weary of itself (Seneca, 2020; Latin text at 57617).

Seneca emphasizes self-hatred and misanthropy more than most later authors do, but his presentation of *taedium* as restless dissatisfaction recurs in modern accounts. For example, Toohey argues that boredom is “characterized by lengthy duration, by its predictability, by its inescapability—by its confinement.” When one is bored, “time seems to stand slow, to the point that you feel as though you stand outside these experiences.” A sense of slow-moving time could be enjoyable, but in boredom, there is “usually a flavor of distaste or, more precisely, of disgust that comes about when one is *satiated* with a situation: so it is that terms such as nausea and biliousness are often used as other names for boredom.” Valuable activities can be boring, but “boredom becomes worse when a situation becomes valueless” (Toohey, 2011, p. −5). Boredom characteristically involves “a feeling of being distanced from one's surroundings and the normal flow of time” (45). The inner state of boredom has observable physical manifestations, such as slumping bodies, drooping necks, yawning, and eyes staring into the distance (35–41). For Toohey, boredom also affords advantages: a heightened sense of “self-perception” and a useful reminder to disengage from “toxic social situations” that are wasting one's time (187, 33).

O'Brien (2014) describes boredom as a “mental state of weariness, restlessness, and lack of interest in something to which one is subjected, which is unpleasant or undesirable, in which the weariness and restlessness are causally related to the lack of interest.” For him, boredom has a volitional aspect: one does not want to engage with or continue with an activity if it is boring. It also has a cognitive aspect: one perceives the activity or object to have features that are boring, such as excessive duration or repetitiveness. An unpleasant combination of weariness and restlessness arises because we are weary with what we are doing, yet restless to do something else. Finally, “In my own experience, boredom is not all that bad–for me and for the people around me. …. Boredom is somewhat bad, but lots of things are worse, and not just a little.”

This mild reaction contrasts with Seán Desmond Healy's account of “hyperboredom,” which he considers endemic in modernity and defines as “an agonizing and chronically painful disease” (Healy, 1984, p. 28). Indeed, empirical studies find cases in which boredom is a global and debilitating condition without any specific target (Fahlman et al., 2013).

Andreas Elpidorou explores psychological research that demonstrates the heterogeneity of boredom as a state and as a trait, its various causes and qualities. He argues, however, that all forms of boredom manifest the same *function*. Boredom reveals that a situation is unsatisfactory and motivates the bored individual to do something purposive and goal-directed. This function is valuable even though the experience itself is to be avoided (Elpidorou, 2021a). Put more strongly, boredom is one of the “elements of a good life” (Elpidorou, 2020, p. 13). A good life does not consist of pure pleasure but is “defined by [the] discovery of personal values and … the formation of one's own commitments” (Elpidorou, 2020, p. 2). Boredom plays an essential role in this process by alerting us to the fact that a given experience is not worthwhile, much as pain alerts us to the presence of a danger or physical damage (Danckert and Eastwood, 2020, p. 54). “The way in which we discover and create values in the world—and develop and grow as human beings—is by having to decide what's interesting and what's not, by being forced to encounter and deal with frustrating situations, and by being asked to figure out what's worth pursuing” (Elpidorou, 2020, p. 160). Thus, one should want to experience boredom at times, rather than doing unworthy activities without feeling bored.

For Heidegger, boredom means interpreting what one experiences as meaningless. However, Heidegger regards the prevailing explanations of life's meaning as false, and thus boredom reveals a truth. Furthermore, Heidegger sees the real meaning of life as temporality, or being aware of the self as passing through time. Boredom enables this awareness by focusing explicit attention on time (cf. Svendsen, 2005, pp. 107–32; Slaby, 2010).

Most scientific researchers would regard their own current mood as irrelevant to their observations of the world, or even as a hindrance that must be overcome before they can conduct valid science. In contrast, Heidegger sees having an “attunement,” such as boredom or anxiety, as an essential and unavoidable way of experiencing anything. Each attunement offers insights, such as boredom's revelation of meaninglessness and temporality. A mood is not exactly subjective, because it offers truths; and it is not exactly objective, because, as it changes, so does the world that we experience. The ultimate truth is that we are creatures that have changing attunements.

Heidegger builds his account of boredom on three successively “profound” examples. In the first, the narrator is bored while waiting for a train “in the tasteless station of some lonely minor railway.” Time, which is usually invisible, painfully drags (Heidegger, 1995, §23a). In the second, the narrator experiences a perfectly pleasant social evening, during which time passes normally. “We come home quite satisfied. We cast a quick glance at the work we interrupted that evening, make a rough assessment of things and look ahead to the next day—and then it comes: I was bored after all on this evening.” Here time does not perceptively drag, yet there is a retrospective appraisal that time was lost and wasted, which hints at insights about the person's whole life (§24b). Third, one makes a judgment without going through the experience at all, as in the general statement: “‘it is boring for one' to walk through the streets of a large city on a Sunday afternoon” (§30). Close inspection of these examples poses the question: “Has man in the end become boring to himself?” (§37).

The proper response is to use boredom to rediscover and embrace the fundamental significance of being, which is temporality. The Russian-American poet Joseph Brodsky made a strikingly similar argument when he told graduating Dartmouth College students:

Boredom … is your window on time's infinity. Once this window opens, don't try to shut it; on the contrary, throw it wide open. For boredom speaks the language of time, and it teaches you the most valuable lesson of your life: the lesson of your utter insignificance. It is valuable to you, as well as to those you are to rub shoulders with. “You are finite,” time tells you in the voice of boredom, “and whatever you do is, from my point of view, futile.” As music to your ears, this, of course, may not count; yet the sense of futility, of the limited significance of even your best, most ardent actions, is better than the illusion of their consequences and the attendant self-aggrandizement (Brodsky, 1995).

In short, boredom reveals a truth. That is not the case, however, with common experiences of boredom; most people who are bored do not attain deep insights. Elpidorou and Freeman (2019) argue that the “profound” boredom identified by Heidegger and Brodsky is not the character trait of being easily or often bored, nor is it the typical state of being bored *by* something. It is an “extraordinary” and “difficult” experience defined by its revelatory power, and as such, it probably will not be detected in statistical studies of populations.

Heidegger's contemporary Adorno analyzes boredom in a sharply different way: as a feature of alienated labor under capitalism. “Boredom is a function of life which is lived under the compulsion to work, and under the strict division of labor. It need not be so.” He acknowledges that he has been “fortunate” to hold a “job, the production of philosophical and sociological works and university teaching,” that grants him autonomy and agency. As a result, he has no interest in “hobbies” or other ways of passing what capitalist society calls “free time,” the hours that are not sold to capital. “I am however well aware that in this I enjoy a privilege, with both the element of fortune and of guilt which this involves: I speak as one who has had the rare opportunity to follow the path of his own intentions and to fashion his work accordingly.” Adorno proposes that whenever people can control their own activity, “boredom rarely figures; it need not figure in activities which cater merely for the desire for pleasure.” However, workers often report boredom even when they're not on the job because the alienation of work “continues to hold people under its spell.” Leisure activities like sports or home improvement turn “free time” into “nothing more than a shadowy continuation of labor” (Adorno, 1969, pp. 162–70).

Adorno dismisses most people's use of their unpaid hours. His framework does not envision civil society or the public sphere as that set of venues in which people voluntarily associate to pray, work, and play (Habermas, 1962; Levine, 2021).

A type of boredom that I have not found described in the philosophical literature involves long periods of time (months or years) in which a person can choose specific activities and events that make time pass so that it is not unpleasant or perceived to drag, yet not enough of perceived value occurs to make the individual feel satisfied with life. Boredom is the subject's appraisal of a whole period of life. This experience is somewhat akin to Heidegger's example of an unsatisfying dinner party, except that Heidegger was soon able to resume interesting intellectual work, which is not available to some people without jobs, or with dull ones.

This species of boredom is, however, prominent in early twenteeth century modernist literature written by women (of whom Virginia Woolf is the most famous), where “boredom can appear as emptiness or deadness, a lack, or simply passive dissatisfaction.” In this feminist fiction, the word boredom “is used, sometimes interchangeably, with a number of other terms defining psychic, spiritual, moral, and physical states in which the self has difficulty accessing authenticity, productivity, and desire—all qualities attributed to one's success as an individual” (Pease, 2012, vii).

It is significant that these women describe women's boredom as dissatisfaction due to a lack of opportunity, whereas Seneca, Heidegger, and other influential male authors have seen it as temporary circumstance that occasions discomfort until it is relieved by satisfying activity or insight.

My own phenomenological account of boredom (meant to be illustrative, not definitive) would emphasize the following features: negative affect; consciousness of the slow passage of time; desire for the current situation to end; lack of curiosity or appreciation. I would not emphasize lack of attention or stimulation, since I perceive myself being bored while attending to things and (frankly) people. I cannot follow Heidegger in seeing boredom as a portal to insight, because that would require embracing his whole philosophy. However, his idiosyncratic analysis illustrates that experiences of boredom vary widely and depend in part on larger frameworks for understanding reality.

## 3. Phenomenology and socially constructed meanings

In general, phenomenology connects an inner experience to a word or phrase that names it. The word in question may have a history of being used in diverse ways. A feeling, such as boredom, that we experience as immediate and direct is socially constructed insofar as it has a name with well-known implications (Goodstein, 2005, p. 4). Changes in the meaning of words may affect our experiences.

Classic phenomenologists sometimes tried to avoid the ambiguous and inconsistent connotations of existing words by coining new ones, which is one source of the difficulty of their texts. Examples of phenomenological coinages include Husserl's *noema* (mental object), Heidegger's *Dasein* (being-there), or Merleau-Ponty's *pensée de survol* (view from above). But one cannot write with neologisms alone. We need phenomenological accounts of widely used words, like “boredom,” in order to reason about how best to use those words.

According to the *Oxford English Dictionary*, relevant meanings of the English terms “to bore” and “boring” are no older than 1750, but the word has since accumulated multiple definitions. This is typical: people redefine words creatively and argumentatively. Heidegger discusses the literal root of the German word *Langeweile:* “long while” (Heidegger, 1995, §19). This etymology will not influence an English-speaker who reflects on being “bored” or a French speaker who experiences *ennui*. The French word may suggest a degree of superiority, since it comes from the Latin *odio*, to hate, as in Horace's famous “*Odi profanum vulgus et arceo*” (“I hate and shun the vulgar crowd”).

It is difficult to reconstruct the experience of boredom before the English word emerged, but it must have been different from today's experience, if only because it was unnamed and lacked conventional moral connotations. Even though people who feel bored today may have similar feelings to human beings thousands of years ago and animals, it is different to call a state or a character “bored” instead of having no word for the category or calling it “ennui” or “acedia” (spiritual apathy). The word “boredom" has rich—and mostly negative—normative connotations that may become part of the experience of the individual or influence institutions, whose assessments and policies then affect individuals. Today, a child who is taught that it is bad to be bored may experience boredom with guilt, resentment, or both. On the other hand, someone who has just read Heidegger might think, “I'm happy that I am bored because I have become aware of time itself, which was concealed while I was interested.” For the latter person, boredom loses its negative affect.

Some prominent authors have argued that boredom accompanies higher intelligence and sensitivity. These claims might encourage a bored person to feel self-satisfied. Others have argued that a wise and sensitive individual cannot be bored, because reality is fascinating if it is properly appreciated. Arthur Schopenhauer and Henry David Thoreau, respectively, represent these arguments (O'Brien, 2022). Either view might affect how we assess ourselves when we notice that we are bored. Does my boredom indicate that I am too sensitive and sophisticated for this situation and the people who seem to be immersed in it, or that I am not smart enough to see what is interesting in it?

Goodstein argues that “modern boredom” has loose connections with older ideas, such as melancholy and *acedia*, but “it can be identified with none of them. … Each of these forms of discontent is embedded in an historically and culturally specific way of understanding human experience—in which I call a *rhetoric of reflection*.” For instance, the pre-modern word “melancholy” assumed that humors may get out of balance: a disease model. *Acedia* implied that the sinner had become estranged from God. Modern boredom—“the experience without qualities”—is “the plague of the enlightened subject, whose skeptical distance from the certainties of faith, tradition, sensation renders the immediacy of quotidian meaning hollow or inaccessible.” Individuals suffering from modern boredom are out of harmony with society and alienated from their “own doing and being” (Goodstein, 2005, 4–10). Modern people who see themselves as bored are liable to be conscious of their individuality and alienation. They might perceive others as also bored: that is a common experience in school. Even so, the individual students are alienated from the institution.

## 4. Normative issues

An argument about whether a mood represents boredom, mere lack of activity, or tranquility is inevitably a question of appraisal. To separate evaluation from phenomenology in an effort to make the latter scientific is a mistake, especially for a context like this special issue. Our whole activity is, and should be, value-laden.

However, there can be a problem of judgmentalism, i.e., unsympathetic attitudes toward people who seem bored. Accusations of boredom can be biased against children and youth, the elderly, or the working class. The accuser may blame the victim for an inhumane or impoverished situation. Calling someone “bored” can overlook important features of that person's experience. Adorno concludes, “If people were able to make their own decisions about themselves and their lives, if they were not caught up in the realm of the eversame, they would not have to be bored. Boredom is the reflection of objective dullness” (Adorno, 1969, p. 161). As I noted earlier, Adorno himself may be biased against seeing the value and satisfaction of working people's civic activity.

Heidegger writes about the boring railway station in the first-person plural: “*We* are sitting. … *We* look at the clock—only a quarter hour has gone by” (Heidegger, 1995, §23a, emphasis added).^1^ The grammar seems inclusive; the reader is expected to be part of the “we.” But the writer happens to be an increasingly famous philosophy professor whose experiences will become more engaging soon after the train ride is over. In short, he is privileged. His class bias emerges in passages like this:

Is not every station boring, even though trains constantly arrive and depart and crowds of people throng? Perhaps it is not only all stations that are boring for us. Perhaps, even though trains constantly enter and leave, bringing people with them, there is still a peculiar sense of something more in these stations which anyone who passes tenement blocks in large cities has experienced. One could say that, while it may be like this for us, some peasant from the Black Forest will take enormous pleasure in it, and therefore boredom is a matter of taste (Heidegger, 1995, §23d).

Evidently, neither the reader nor the author lives in a tenement house or identifies as a peasant. Since academic research is, almost by definition, conducted by people who hold currently bourgeois roles (albeit sometimes precarious ones), it is crucial not to let first-person plural phenomenology supplant social science. Researchers and professors need to learn what boredom feels like to other kinds of people. In particular, what about people who are diverted moment to moment but who feel that their life lacks memorable activities and events?

Danckert and Eastwood offer advice about responding to boredom, based primarily on empirical research about individual psychology. For instance, “Seek out activities that clarify, rather than obscure, your desires and goals. Pursue goals that give expression to your values—things that matter to you. Do things for their own sake, rather than as a means to avoid something else” (Danckert and Eastwood, 2020, p. 204). Their exemplars include a factory worker who finds interest in a lifetime of repetitive labor and another who invents a machine so that he can quit his task (Danckert and Eastwood, 2020, p. 33–58). These are individuals adjusting to social circumstances. This approach gives little attention to social critique or to the possibility of restructuring institutions like factories and schools so that they are more meaningful. In contrast, Elpidorou argues that boredom is unjust—and sometimes “cruel” —“because some groups are disproportionately impacted by boredom through no fault of their own” (Elpidorou, 2021b, p. 193, 172). If, for individuals, being bored is a useful sign that an activity lacks value and a spur to change what we are doing, then for a society, pervasive boredom in some situations (factories or offices, schools, or homes) should perhaps be a spur to change the conditions that create those situations (Ros Velasco, 2021, p. 303). This was Adorno's point but is “less traveled terrain” today, when much of the literature focuses on individuals' choices (Todman, 2021, p. 139).

Whether boredom is wrong surely depends on what one is bored of. Schopenhauer, Heidegger, Healy some others have been interested in boredom about life as a whole. They think life—or modern life—essentially merits boredom, and this realization leads to wisdom. Others may be more concerned when people are bored of specific things that should interest them, such as the subjects that are taught in school, or the news. Still others are concerned that people are bored because of boring experiences, such as bad pedagogy or “bullshit jobs” (Graeber, 2018). Adorno essentially describes all jobs under capitalism that way (Adorno, 1969). We can be bored of other people, and that often seems like a way of undervaluing those individuals. But sometimes a person who thoughtlessly exploits our time is responsible for our being bored.

When people are bored of something that we think should interest them, we can consider at least three possible remedies: changing their attitude, changing the way the object is presented to them, or changing the object itself.

For instance, formal politics (elections and lawmaking) bores many people. Theorists in the long republican political tradition believe that politics merits everyone's attention (Arendt, 1963). That can be an argument for encouraging or scolding people not to be bored by politics, or teaching and presenting politics in less boring ways, or changing political processes so that they are more interesting. For example, Josh Lerner notes that electoral processes typically violate principles well known to game-designers, such as maintaining a chance for every player to win until the very end. Alternative formats, such as Participatory Budgeting, are less boring because they follow these design principles (Lerner, 2014). Instead of teaching citizens to be interested in tedious processes, Lerner would make the political system more “fun” (his keyword). The problem of boredom in school has generated a similar debate (Healy, 1984, pp. 118–140).

Since ancient times, authors who have decried boredom have typically recommended either of two paths for the individual: (1) purposive activity or (2) appreciation and mindfulness (Seneca, 2020). The former treats boredom as close to apathy and laziness and recommends doing something new. The latter treats it as primarily about inattention and recommends noticing what is already happening—such as one's breath, the passage of time, or the needs of the person who is talking.

Kieran Setiya offers a way to think about “living in the moment.” His argument rests on a distinction between *telic* activities, which we conduct in order to accomplish them, and *atelic* activities, which we do for their own sake. “Cook[ing] dinner for your kids, help[ing] them finish their homework, and put[ting] them to bed” are “telic activities through and through”: aimed at their accomplishment. On the other hand, “parenting is complete at every instant; it is a process not a project” (Setiya, 2017, p. 141).

Modern capitalism promises atelic opportunities, from playing golf in retirement to purchasing a yoga class. These are, however, relatively marginal, inaccessible to many people, and not directly helpful for improving the world. Their availability only encourages us to view our required activities as boring. “In staving off boredom by finding things to do, you have condemned yourself to misery” (Setiya, 2017, p. 132). In contrast, some classical texts recommend viewing every activity as purely atelic. Setiya quotes Krishna from the Laurie L. Patton's translation of *Baghavad Gita*: “Motive should never be in the fruits of action, / nor should you cling to inaction. … / Let go of clinging, and let fulfillment / and frustration be the same.” However, Setiya claims that what we accomplish—not only our attitude toward it—is important.

His advice is to view the telic as atelic. Strive to put the children in bed (and do that as well as you can), but also think of yourself as parenting. Attend meetings, write emails, and perform calculations all day, but also see yourself as playing a worthy social role. We may be able to redescribe what we are doing in purposive terms. This reinterpretation could, to quote Wordsworth, have “the power to make/Our noisy years seem moments in the being / Of the eternal Silence” (Wordsworth, 1884). However, Setiya mostly discusses bourgeois work and household activities, whereas work that is degrading and obligatory is surely harder to view as atelic.

## 5. Conclusion

Given the complexity of the definition and the range of contexts, it seems unwise to adopt a uniform attitude toward boredom. However, we should be explicitly normative, i.e., we should ask whether boredom is right or wrong under the specific circumstances. We should ask whether the problem is the situation or the person who is bored; in principle, either one could be changed. If the individual should change, we should ask whether the bored person should do something different or change their attitude toward what they are already doing. Finally, we should not neglect the possible virtues of the state of boredom, particularly the access that it might afford to truths.

## Author contributions

The author confirms being the sole contributor of this work and has approved it for publication.

## Footnotes

^1^“*Wir sitzen z. B. auf einem geschmacklosen Bahnhof einer verlorenen Kleinbahn…*” (Heidegger, 1983, p. 140).
